# Broader Perspective on Atherosclerosis—Selected Risk Factors, Biomarkers, and Therapeutic Approach

**DOI:** 10.3390/ijms25105212

**Published:** 2024-05-10

**Authors:** Piotr Fularski, Witold Czarnik, Bartłomiej Dąbek, Wiktoria Lisińska, Ewa Radzioch, Alicja Witkowska, Ewelina Młynarska, Jacek Rysz, Beata Franczyk

**Affiliations:** 1 Department of Nephrocardiology, Medical University of Lodz, ul. Zeromskiego 113, 90-549 Lodz, Poland; 2 Department of Nephrology, Hypertension and Family Medicine, Medical University of Lodz, ul. Zeromskiego 113, 90-549 Lodz, Poland

**Keywords:** atherosclerosis, risk factors, biomarkers, novel treatment

## Abstract

Atherosclerotic cardiovascular disease (ASCVD) stands as the leading cause of mortality worldwide. At its core lies a progressive process of atherosclerosis, influenced by multiple factors. Among them, lifestyle-related factors are highlighted, with inadequate diet being one of the foremost, alongside factors such as cigarette smoking, low physical activity, and sleep deprivation. Another substantial group of risk factors comprises comorbidities. Amongst others, conditions such as hypertension, diabetes mellitus (DM), chronic kidney disease (CKD), or familial hypercholesterolemia (FH) are included here. Extremely significant in the context of halting progression is counteracting the mentioned risk factors, including through treatment of the underlying disease. What is more, in recent years, there has been increasing attention paid to perceiving atherosclerosis as an inflammation-related disease. Consequently, efforts are directed towards exploring new anti-inflammatory medications to limit ASCVD progression. Simultaneously, research is underway to identify biomarkers capable of providing insights into the ongoing process of atherosclerotic plaque formation. The aim of this study is to provide a broader perspective on ASCVD, particularly focusing on its characteristics, traditional and novel treatment methods, and biomarkers that can facilitate its early detection.

## 1. Introduction

Atherosclerotic cardiovascular disease (ASCVD) continues to stand as the foremost contributor to mortality on a global scale [1]. At the foundation of ASCVD lies atherosclerosis, which can affect various arterial vessels within the body, narrow them, and manifest itself in both acute and chronic forms. Among acute conditions are myocardial infarction (MI), stroke, acute mesenteric ischemia, acute peripheral arterial occlusion, or even thoracic aorta rupture. The chronic group encompasses, amongst others, recurrent transient ischemic attacks (TIAs), stable angina, aortic aneurysm, worsening renal functioning, or chronic limb ischemia [2]. Atherosclerosis develops through three consecutive phases, starting from the lipid-streak phase, via the fibrous plaque phase, to the final phase, namely, advanced lesions with thrombosis. At the outset, lipids are retained and trapped inside the intima of the arterial wall, leading to macrophage infiltration and the absorption of surplus lipids, resulting in foam cells formation. The next phase entails the passage of vascular smooth muscle cells (VSMC) to establish a durable fibrous cap, covering the atherosclerotic area. Overabundance of foam cells, however, results in necrosis inside the plaques and triggers the necrotic core formation. As it grows, the fibrous cap may rupture and expose the necrotic core, bringing it into contact with blood, thereby provoking thrombosis [3]. Different types of plaques vary in their susceptibility to rupture. The propensity for rupture primarily depends on the composition of the atherosclerotic plaque itself. Inflammatory, lipid-rich plaques appear to be decidedly more prone to rupture than those covered by a thick fibrous cap. Of course, the size of the lesions also matters, although it holds secondary significance compared to their structure [4]. Undoubtedly, the primary, although not sole, factor contributing to atheromatous plaques formation is the elevated level of low-density lipoprotein cholesterol (LDL-C) in the bloodstream [5]. The level of LDL-C is particularly elevated in familial hypercholesterolemia (FH), which results in a significant acceleration of ASCVD development [6]. Other diseases significantly contributing to the development of ASCVD include hypertension and diabetes mellitus (DM) [7]. DM also contributes to the development of another pathology significantly impacting atherosclerosis, namely, the development of diabetic nephropathy. This condition, in turn, can decrease glomerular filtration rate (GFR) and may result in chronic kidney disease (CKD), even leading to end-stage renal disease (ESRD) [8]. CKD, in turn, is responsible for the retention of uremic toxins, increased oxidative stress, vascular calcification, and notably, chronic inflammation. All of these factors exacerbate the progression of ASCVD [9]. Particularly, the role of inflammation is increasingly emphasized in the context of the pathogenesis of atherosclerosis [10]. The correlation between chronic inflammatory state and increased frequency of premature atherosclerosis becomes evident, among others, in individuals suffering from rheumatoid arthritis [11]. Similarly, in individuals with viral hepatitis C, an increased frequency of ASCVD complications has been observed [12]. However, the etiology is even more intricate. It appears that disruptions within the gut microbiota can also play a significant role in the pathogenesis of this entity. Bacteria inhabiting the gastrointestinal tract produce metabolites that subsequently participate in cholesterol homeostasis [13]. In the context of ASCVD etiology, one cannot overlook the role of factors such as obesity, inadequate diet, sleep deprivation, air pollution, active and passive smoking, and a lack of physical activity [14,15]. It has also been noted that atherosclerosis affecting coronary arteries typically manifests more prominently in men than in women [16].

**Figure 1:**
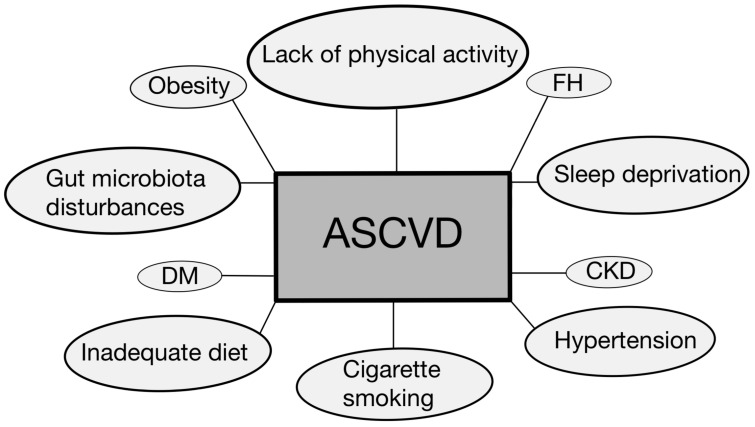
Selected disease entities and other risk factors contributing to the development and progression of ASCVD [6,7,9,13,14,15]. ASCVD—atherosclerotic cardiovascular disease; CKD—chronic kidney disease; DM—diabetes mellitus; FH—familial hypercholesterolemia.

Selected risk factors for atherosclerosis are depicted in Figure 1. Moreover, given the complexity, prevalence, and risk associated with this disease, our review delves into the topic of biomarkers and modern treatment approaches.

## 2. Selected Biomarkers

Atherosclerosis is defined as a persistent inflammatory condition of the artery wall [17]. Neo-angiogenesis is significantly engaged in plaque instability, resulting in plaque rupture. Vascular endothelial growth factor (VEGF), which promotes cell proliferation, inhibits apoptosis, increases vascular permeability, vasodilation, and recruits inflammatory cells to the injury site, contributes to the development of atherosclerosis and worsens cardiovascular disease (CVD) [18]. The landscape of cardiovascular research is rapidly advancing, with the quest to pinpoint and authenticate biomarkers for atherosclerosis taking center stage. This pursuit is driven by the promise that such biomarkers hold not only for deepening our grasp of the disease’s intricate biological foundations but also for steering individualized treatment approaches. Among the myriad biomarkers scrutinized, those linked to inflammation, lipid metabolism, and genetic predispositions stand out for their capacity to foretell the development and escalation of atherosclerosis [19]. Particularly, C-reactive protein (CRP) has emerged as a key inflammatory biomarker, its elevated levels closely correlating with an increased risk of cardiovascular events. This correlation underscores the critical role of inflammation in the progression of atherosclerosis. Findings from recent investigations reveal that intensive statin therapy can significantly reduce both LDL cholesterol and CRP levels, leading to a marked decrease in cardiovascular incidents and mortality rates. The most pronounced benefits of such treatment were observed in individuals who achieved the lowest levels of LDL cholesterol and CRP, highlighting the dual importance of managing cholesterol levels and inflammatory processes in cardiovascular risk reduction. Yet, it is important to note that the decrease in CRP levels following statin therapy may reflect a reduction in LDL-induced inflammatory activity within atherosclerotic plaques, rather than a direct effect on the CRP pathway itself [20]. High-sensitivity C-reactive protein (hsCRP), while a recognized indicator of CVD risk, especially following acute coronary syndrome (ACS), has evolved into a metric of residual inflammatory cardiovascular risk, akin to the role played by LDL-C. This evolution expands the understanding of cardiovascular risk to include inflammatory factors. Despite the acknowledged clinical relevance of hsCRP, its role as a potential target for specific anti-inflammatory treatments remains debated. Consequently, research has also explored other inflammatory mediators, positioning hsCRP more as a marker of underlying inflammation rather than a direct target for therapy. This nuanced approach to cardiovascular risk assessment and management underscores the complexity of atherosclerosis as a disease influenced by both lipid accumulation and inflammatory processes [21].

### 2.1. Inflammasome and Cytokines

The NOD-like receptor protein 3 (NLRP3) inflammasome participates in the development of AH [22]. DAMPs (damage-associated molecular patterns), such as LDL, cholesterol crystals, calcium pyrophosphate crystals, uric acid crystals, hyperglycemia, disrupted blood flow, and hypoxia [23], activate the NLRP3 inflammasome, which produces interleukin-1β (IL-1β) via caspase-1-mediated cleavage of pro-IL-1β. The NLRP3 inflammasome is a multimeric cytosolic protein complex composed of pathogen-associated molecular patterns (PAMPs), DAMPs, and neutrophil extracellular traps (NETs) [24], which are pro-atherosclerotic, cytotoxic, nucleus-derived, and net-like chromatin structures released extracellularly. In response to infections and inflammatory stimuli such as cholesterol crystals, oxidized low-density lipoprotein (oxLDL), oxysterols, platelets, and chemokines, neutrophils release cytosolic and nuclear material, forming a net-like extracellular structure [25]. This also activates the NLRP3 inflammasome [26]. During pyroptosis, the NLRP3 inflammasome generates IL-1β and IL-18, which are strong systemic inflammatory mediators. IL-1β stimulates the production of both itself and IL-6. IL-1β is initially generated as pro-IL-1β, which is then cleaved into its active form by caspase-1 once the NLRP3 inflammasome is activated [27]. IL-1β has autocrine, paracrine, and endocrine actions and is linked to the development of autoinflammatory illnesses, gout, diabetes, atherosclerosis, and neurodegenerative disorders. IL-1β stimulates its own synthesis, along with that of IL-6, which is a hormone-like cytokine that contributes to innate and adaptive immunity and has both pro- and anti-inflammatory characteristics. Production of IL-6 takes place in T cells, which are the primary producers of IL-6, as well as monocytes, fibroblasts, and endothelial cells [28]. Apart from inflammation, IL-6 plays a metabolic role, regulating lipid metabolism and insulin resistance [27]. IL-6 also interacts with soluble or membrane-bound receptors, as well as glycoprotein 130 (gp130), forming a hexameric complex. When a receptor is activated, intracellular signaling begins via the Janus kinase and signal transducer and activator of transcription (JAK-STAT) pathways, eventually leading to target gene transcription. This causes both local and systemic inflammation, which results in disruption of the balance of regulator and effector T and B cells, as well as immunoglobulin and acute-phase protein synthesis [29]. In turn, IL-6 stimulates the liver to produce acute phase reactants such as plasminogen activator inhibitor-1 (PAI-1) and fibrinogen, both of which have thrombogenic qualities. In addition, IL-6 stimulates apolipoprotein(a) [apo(a)] production by activating the LPA gene’s promoter region. Other variables that can activate IL-6 include TNF (tumor necrosis factor), toll-like receptors, prostaglandins, adipokines, and stress. During acute inflammation, IL-6 increases the hepatic production of several acute-phase proteins, including fibrinogen, PAI-1, serum amyloid A (SAA), and CRP, which are implicated in host immunological responses as well as thrombogenesis. It is worth noting that patients with COVID-19 have a higher risk of MACE due to IL-6 [30]. Persistently high IL-6 levels lead to chronic inflammation and, ultimately, tissue damage. CCs, the primary causes of atherosclerosis, are currently regarded as the most critical trigger for NLRP3 inflammasome activation. This event is undesirable because it can cause lipid peroxidation and affect the physiological activity of bio enzymes. Oxidized phospholipids are known to promote inflammation. As a result, oxLDLs are used as a clinical indicator of plaque inflammation. The mechanisms of endothelial dysfunction reveal that inflammatory factors play an important role in the pathophysiology of this condition [31]. Modified lipids cause the intima’s inflammatory cells to release chemokines and cytokines such as TNFα, IL-1, -4, and -6, and interferon-gamma. These chemokines and cytokines subsequently activate additional leukocytes, endothelial cells, and adhesion molecules, including vascular cell adhesion molecule-1 (VCAM-1) [32]. These altered lipoproteins contribute to the formation of atherosclerotic plaques. LDL’s cholesterol-rich lipoproteins are absorbed and integrated into macrophages, and they also produce ROS and reactive nitrogen species (RNS), pro-oxidants that contribute to the development of atherosclerosis. Increased amounts of ROS may be induced by reduced mitochondrial activity, which is associated with aging. The mechanisms of endothelial dysfunction reveal that inflammatory factors play an important role in the pathophysiology of this condition. The intima of a damaged arterial wall is rich in lymphocytes and mast cells [33]. Modified lipids cause the intima’s inflammatory cells to release chemokines and cytokines such as TNFα, interleukin-1, -4, and -6, and interferon-gamma. These chemokines and cytokines subsequently activate additional leukocytes, endothelial cells, and adhesion molecules, including VCAM-1. These altered lipoproteins contribute to the formation of atherosclerotic plaques. LDL’s cholesterol-rich lipoproteins are absorbed and integrated into macrophages, and they also produce ROS and RNS, pro-oxidants that contribute to the development of atherosclerosis. Increased ROS levels might be driven by decreased mitochondrial activity, which is linked to aging [31]. OxLDLs agitate endothelial cells, increasing the production of adhesion-forming chemicals. The ROS and RNS convert LDL-C into ox-LDLs, which form part of the intimal layer [33]. Atherosclerosis begins with the subendothelial retention of ApoB 100, which contains lipoproteins. LDL penetration across a defective endothelium involves a number of variables. The process can be divided into three phases: commencement, progression, and thrombosis. The intima is bordered by a single layer of endothelial cells known as the endothelium and a subendothelial extracellular matrix, which is composed of collagen and elastin. Endothelium regulates vascular tone, coagulation, and maintains vascular homeostasis through highly controlled systems, including nitric oxide, prostacyclin, and endothelin-1 [34]. Tunica media include a large number of smooth muscle cells (SMCs), which are organized concentrically inside an elastin-rich cellular matrix to store the kinetic energy necessary for pulsatile flow transmission. The adventitia is made up of mast cells, fibroblasts, and a proteoglycan and collagen-rich matrix. The internal and external elastic laminas divide the intima, media, and adventitia, respectively. Nonetheless, the artery is damaged by the interaction of Ox-LDL and other risk factors, hardening and narrowing the lumen, resulting in disrupted blood flow [5]. Fibrinogen has been established as a significant independent risk factor for CVD. Fibrinogen has also been linked to classic cardiovascular risk factors, implying that fibrinogen elevation might be a mechanism by which these risk factors work. There are various ways in which fibrinogen might raise cardiovascular risk. First, it attaches to active platelets via glycoprotein IIb/IIIa, which promotes platelet aggregation [35]. Second, higher fibrinogen levels encourage fibrin production. Third, it makes a significant contribution to plasma viscosity. Finally, it is an acute-phase reactant that increases in inflammatory conditions [36].

### 2.2. Fibroblast Growth Factor-23

Fibroblast growth factor-23 (FGF-23), a hormonal activator of urinary phosphate excretion, rises in blood concentration during early CKD [37]. FGF-23’s activities are mainly restricted to organs that express the coreceptor Klotho, notably the renal tubules, where it inhibits sodium phosphate cotransporters. Animal studies indicate direct (i.e., Klotho-independent) [38] cardiotoxicity, leading to suggestions that FGF-23 should be viewed not just as a marker for CVD but also as a causative contributing element. A meta-analysis of traditional epidemiological research revealed independent relationships between greater circulating FGF-23 concentrations and an increased risk of atherosclerotic cardiovascular illnesses (MI and stroke) and heart failure (HF). However, there is significant ambiguity about causation because these relationships lack a strong “exposure-response” link and are non-specific; there are additional reports of positive correlations between FGF-23 and infection, fractures, acute kidney injury (AKI), and all-cause mortality-16. Thus, residual confounding remains a plausible explanation for these FGF-23 relationships [39]. Naturally occurring genetic variations (single nucleotide polymorphisms [SNPs]) related with biological features are assigned at random during conception and can be utilized as tools in genetic epidemiology studies. This Mendelian randomization (MR) strategy can overcome some of the constraints inherent in traditional observational studies [40], and it is especially useful when attempting to account for confounding by kidney function. Previous MR investigations of FGF-23 have been hampered by low power since the genetic variations employed explain only about 3% of the diversity in FGF-23, and they have not studied the breakdown of correlations with atherosclerotic versus non-atherosclerotic phenotypes [38].

### 2.3. GDF-15

In atherosclerosis, which is a chronic inflammatory condition characterized by the monocytes activity in lipid deposition within vessel walls [41]. These monocytes, upon ingestion of lipoproteins like oxLDLs, transform into macrophages and give rise to foam cells. Notably, there exists a reciprocal relationship between oxLDLs and growth and differentiation factor-15 (GDF-15): oxLDLs stimulates GDF-15 production in macrophages, while GDF-15 inhibits lipid accumulation induced by oxLDL and modulates the pro-inflammatory cytokine profile of vessel wall macrophages. GDF-15 also influences lipid metabolism, crucial in atherosclerosis, by reducing lipid accumulation and foam cell formation, potentially through activation of the peroxisome proliferator-activated receptor β/δ (PPARβ/δ) pathway [42].

Moreover, GDF-15 appears to influence lipid synthesis independently of oxLDL in human macrophages, suggesting a novel role in atherosclerotic plaque formation and progression by disrupting autophagic processes, impacting lipid homeostasis. While these findings suggest a potential atheroprotective effect of GDF-15, clinical studies have linked it to increased cardiovascular event risk and mortality in individuals with established ASCVD. GDF-15 contributes to both the onset and advancement of atherosclerosis [37,42].

Studies investigating GDF-15 in coronary artery disease (CAD) have shown its potential as a predictive biomarker for acute coronary syndrome (ACS) recurrence, MI-related complications, HF, and mortality. In combination with N-terminal pro-B-type natriuretic peptide (NT-proBNP), GDF-15 can identify high-risk patient groups. Furthermore, research on the relationship between GDF-15 and coronary artery calcium score (CACS) indicates a significant positive correlation, highlighting its potential as a diagnostic marker in CAD [42].

### 2.4. Exosomes

Exosomes (EX) are extracellular vesicles that originate within the endosomes of eukaryotic cells [43]. These vesicles comprise a diverse array of molecules, characterized by their aqueous core surrounded by a lipophilic membrane. The biosynthesis of EX involves the incorporation of proteins, DNA, RNA, and lipids from the donor cell’s membrane into the multivesicular bodies, subsequently leading to the formation of EX. This process culminates in the secretion of EX upon fusion with the parent cell’s membrane [44]. Notably, exosomes are distinguished by their significant content of microRNAs (miRNA) [45], alongside substantial quantities of long non-coding RNAs (lncRNA) and circular RNAs (circRNAs) [46]. These nucleic acid components have been implicated in the pathogenesis of atherosclerosis, highlighting the pivotal role of exosomes in cellular communication and disease progression.

#### 2.4.1. miRNA

As discussed above, platelet activation and endothelial damage play essential roles in atherosclerosis pathogenesis. With the usage of immunohistochemistry and western-blotting technique, Li et al. established that the levels of miRNA-223, miRNA-339, and miRNA-21 were elevated in thrombin-activated platelet exosomes. Additionally, miRNA-223 was established to be a key player in the inhibition of the phosphorylation of p38 mitogen-activated protein kinases (p38), c-Jun N-terminal kinases (JNKs), and extracellular signal-regulated kinases (ERK) and blocked the nuclear translocation of NF-κB p65. Furthermore, the miR-223 inhibitor blocked the down-regulating effects of exosomes on ICAM-1 expression [47]. The MAPK pathway, encompassing ERK1/2, JNK, and p38 MAPK, represents a fundamental signaling mechanism that connects cell surface receptors to critical regulatory targets within the cell, showcasing its evolutionary conservation and versatility. This pathway is pivotal in orchestrating the inflammatory response, becoming activated in response to a plethora of stimuli such as oxidative stress, cytokines, and growth factors. These stimuli are particularly abundant within the milieu of atherosclerotic lesions, indicating the pathway’s crucial role in the disease’s progression. The interaction between miRNAs and the MAPK pathway elucidates a sophisticated regulatory network, where miRNAs not only modulate the activity of this pathway but also influence the overall inflammatory environment characteristic of atherosclerosis. Such insights into the molecular dynamics of atherosclerosis offer promising avenues for the development of targeted therapies aimed at mitigating the inflammatory processes at the heart of this CVD [48].

miRNAs derived from exosomes, isolated seamlessly from various fluids, represent a leap forward in biomarker development for diseases like atherosclerosis. Their superiority over circulating miRNAs stems from the ability to purify exosomes from specific cell types, thereby enhancing the biomarker’s sensitivity and specificity [49]. Recent studies have illuminated the promise of specific exosomal miRNA profiles, such as miR-122-5p, miR-27b-3p, and miR-101-3p, in forecasting recurrent ischemic events in cases of intracranial atherosclerosis [50]. Furthermore, the detection of certain exosomal miRNAs, including miR-92a-3p and miR-30e, has been linked to atherosclerotic conditions, suggesting their utility in diagnosing and managing coronary atherosclerosis [51,52]. While these findings underscore the diagnostic and prognostic potential of exosomal miRNAs in atherosclerosis, the transition of these biomarkers into clinical practice awaits further validation in extensive cohort studies and the standardization of isolation methods as per international guidelines.

#### 2.4.2. lncRNA

lncRNAs have garnered significant attention for their pivotal roles in regulating essential cellular mechanisms. Characterized by their length of over 200 nucleotides and possessing mRNA-like features such as 5′ capping, splicing, and polyadenylation, lncRNAs have demonstrated their capacity to interact with various molecular entities, including RNA, DNA, proteins, and RNA-binding proteins [53]. Among these, the macrophage-specific lncRNA MAARS (macrophage-associated atherosclerosis lncRNA aequence) has emerged from RNA-seq profiling of the intima of lesions, revealing a remarkable 270-fold increase in expression in the aortic intima during atherosclerotic progression and a 60% decrease upon regression. Intriguingly, MAARS knockdown in LDLR-/- mice leads to a 52% reduction in atherosclerotic lesion formation, a process that is largely independent of lipid profiles and inflammatory responses. Instead, this reduction is attributed to decreased macrophage apoptosis and enhanced efferocytosis within the vessel wall. The interaction between MAARS and HuR/ELAVL1, a key RNA-binding protein involved in apoptosis regulation, underscores MAARS’s crucial role in macrophage survival mechanisms, further affecting the expression of significant apoptosis and cell-cycle regulators such as p53, p27, Caspase-9, and BCL2 through HuR cytosolic shuttling [54].

Additionally, the landscape of lncRNAs in vascular biology is further enriched by the discovery of lncRNA NEXN-AS1, which influences endothelial cell activation and monocyte adhesion through the TLR4/NF-κB signaling pathway, acting as a deterrent to atherogenesis [55]. Moreover, lncRNA CCL2 is highlighted for its contribution to human atherosclerosis by upregulating CCL2 mRNA levels in endothelial cells, pointing to a complex network of lncRNA-mediated regulation in the vascular system [56]. These discoveries not only elucidate the multifaceted roles of lncRNAs in vascular disease states but also pave the way for innovative therapeutic strategies targeting these non-coding RNAs to combat atherosclerosis and related conditions.

#### 2.4.3. circRNA

Circular RNAs (circRNAs) represent a novel class of RNA molecules characterized by their unique closed-loop structure, resulting from a process known as back-splicing, where a covalent bond links the ends of linear RNA sequences [57]. These non-coding RNAs play crucial roles in the regulatory mechanisms governing protein transcription, functionality, and even the translation process leading to polypeptide formation [57]. An array of research endeavors has delved into the exploration of circRNAs, with six in vitro studies shedding light on the upregulation of circCHFR within atherosclerotic conditions, achieved through the stimulation of cells using oxLDL or PDGF. This upregulation has been linked to the sponging of miRNAs and the overexpression of genes conducive to atherosclerotic processes. For example, the sequestration of miR-370 was observed to enhance the expression of FOXO1/Cyclin D1, thus promoting the proliferation and migration of VSMCs [58]. In contrast, research conducted by Zhang W-B et al. revealed diminished levels of circHIPK3 in both the serum and tissues of patients with atherosclerosis, correlating with increased osteogenic and chondrogenic differentiation, as well as elevated mineralization and calcium deposition in VSMCs in vitro. Notably, the overexpression of circHIPK3 was found to engage in miR-106a-5p sponging, leading to the activation of the MFN2 gene. This activation played a pivotal role in mitigating osteogenic and chondrogenic differentiation, thereby reducing calcium buildup in VSMCs [59,60,61,62].

### 2.5. Immune Cells

Mast cells have been confirmed to exist within atherosclerotic lesions, with studies demonstrating their ability to initiate the phosphorylation of p38 MAPK upon stimulation by oxLDL) under laboratory conditions [63]. OxLDL plays a critical role as a pro-inflammatory and pro-atherogenic agent, impacting all phases of atherosclerosis by promoting the release of cytokines and chemokines in macrophages, VSMCs, and endothelial cells [64]. Furthermore, clinical research has highlighted the significance of serum oxLDL levels as a key indicator for the severity of acute coronary syndrome. Notably, research by Huang et al. that focused on younger patients, specifically those under 55 years of age, demonstrated in a study involving 128 CAD patients that oxLDL is a significant risk factor for the onset of atherosclerosis, independent of smoking, hypertriglyceridemia, and the ApoB/ApoA1 ratio. The level of oxLDL was significantly higher in the CAD group than in control (*p* < 0.01) [65]. Additionally, it was found that serum levels of oxLDL are affected by smoking habits, with smoking noted to increase these levels [66]. Therefore, the serum level of oxLDL might be a valuable independent atherosclerosis predictor in the younger population.

T lymphocytes play a critical role in the progression of atherosclerosis, with their association observed in calcific nodules within the fibrous cap and the plaque itself [67]. These cells infiltrate lesions at an early stage, likely drawn by pro-inflammatory cytokines released by macrophages, smooth muscle cells (SMCs), and valvular interstitial cells (VICs), and their migration into the tissue is aided by adhesion molecules like VCAM-1, ICAM-1, and P-selectin on activated endothelial cells. As the disease progresses, neo-angiogenesis offers additional pathways for T lymphocyte infiltration [68]. The involvement of diverse T cell subsets, including CD4+ helper and CD8+ cytotoxic T cells, in atherosclerosis is well-documented [68,69]. Notably, these subgroups exhibit dual roles in disease progression, serving as both pro- and antiatherogenic factors, which positions them as potential biomarkers for atherosclerosis. TH1 cells, a subtype of T helper cells, are particularly significant for being the most common T cells in atherosclerotic plaques and are known to exacerbate inflammation and plaque instability [70]. This is supported by findings that a genetic deficiency in the TH1-specific transcription factor TBX21 or IFN-γ reduces atherosclerosis in hyperlipidemic mice, underscoring their pro-atherogenic influence [71,72]. On the other hand, TH17 cells, activated by IL-17, are implicated in plaque development, with studies showing a decrease in aortic plaque formation in IL-17 deficient mice, highlighting the complexity of T cell roles in atherosclerosis [73]. The exact impact of CD8+ T cells remains elusive, with some evidence pointing to a pro-atherogenic role in the early stages of plaque development, yet definitive conclusions about their overall contribution to atherosclerosis are still being explored [74,75].

The complex interplay between immune responses and atherosclerosis is significantly influenced by the roles of natural killer T (NKT), which interact with lipid antigens and chemokines within atherosclerotic environments. NKT cells, known for their ability to identify lipid antigens presented on CD1d molecules by antigen-presenting cells, are implicated in the aggravation of atherosclerosis [76]. Their presence is notably marked in atherosclerotic lesions where they contribute to inflammation through the secretion of cytokines and engage in processes like neo-angiogenesis, which destabilizes plaques [77]. This involvement is primarily due to their secretion of granzyme B, perforin, and IL-8, the latter of which promotes angiogenesis through the induction of EGFR in endothelial cells [78]. The exacerbation of atherosclerosis by NKT cells is evidenced in studies utilizing ApoE-/- mouse models, demonstrating the cells’ pro-atherogenic activities and their contribution to disease progression in cardiovascular tissues [79].

**Figure 2:**
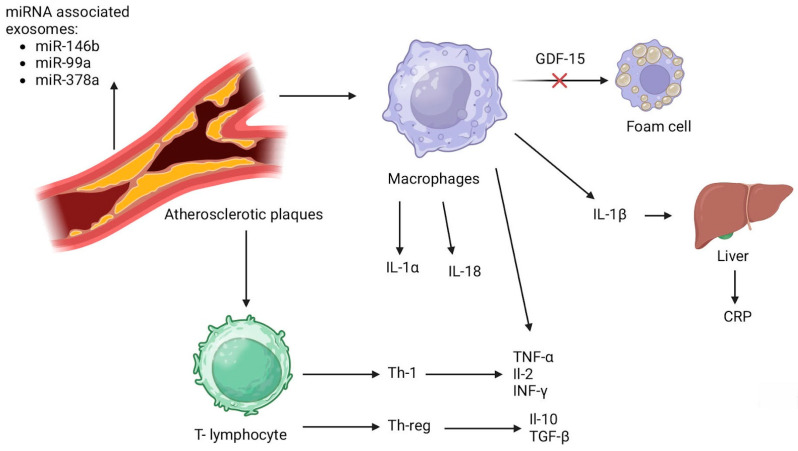
Inflammatory cascade of markers [41,50,80]. IL-1α—interleukin 1α; IL-18—interleukin 18; IL-1β—interleukin 1β; CRP—C-reactive protein; Th-1—type 1 helper T- cell; Th-reg—regulatory T- cell; TNF-α—tumor necrotic factor α; IL-2—interleukin 2; INF-γ—interferon γ; IL-10—interleukin 10; TGF-β—transforming growth factor β; GDF-15—growth differentiation factor 15.

The exploration of NKT cells has expanded to understand their activation by both endogenous self-lipid and exogenous microbial lipid antigens. This activation prompts a swift cytokine and cytotoxic protein response, pivotal in the pathogenesis of atherosclerosis. The modulation of NKT cell activity by gut microbiota illustrates the dynamic interaction between host immunity and microbial factors in cardiovascular disease. Parallel to NKT, cells are attracted and activated by specific chemokines such as MCP-1 and fractalkine. These chemokines not only facilitate NKT cell migration into the lesion site but also enhance their cytotoxic function and IFN-γ production, further promoting a pro-atherogenic environment. The cytokines capable of recruiting and activating NK cells, including IL-15, IL-12, IL-18, and IFN-α, have been identified to contribute to atherosclerosis, highlighting the integral role of NK cells in the disease’s development [80]. This comprehensive view underscores the critical contribution of NKT cells to the inflammatory processes central to atherosclerosis. By delineating the pathways through which these cells influence disease progression, research continues to unravel the potential for targeted interventions aimed at modulating immune cell activity in atherosclerosis [81]. The inflammatory cascade of markers has been shown at Figure 2 [41,50,80].

## 3. Treatment Possibilities

### 3.1. Statins and Ezetimibe

Over the past three decades, statins have played a significant role in preventing numerous cardiovascular events associated with atherosclerosis and reducing cardiovascular mortality [82]. Statins function by reducing the amount of cellular cholesterol through targeted inhibition of the enzyme HMG-CoA (3-hydroxy-3-methylglutaryl-coenzyme A) reductase. This inhibition curtails the synthesis of cholesterol, consequently diminishing hepatic cholesterol levels. As a result, there is an upregulation of LDL-receptors on the membranes of liver cells, facilitating the removal of LDL-C particles from the bloodstream [83]. Additional effects of statins, which are not completely elucidated but are noteworthy, include LDL-independent actions, also known as pleiotropic effects. They are shown in the Table 1 below [84,85,86].

**Figure 3:**
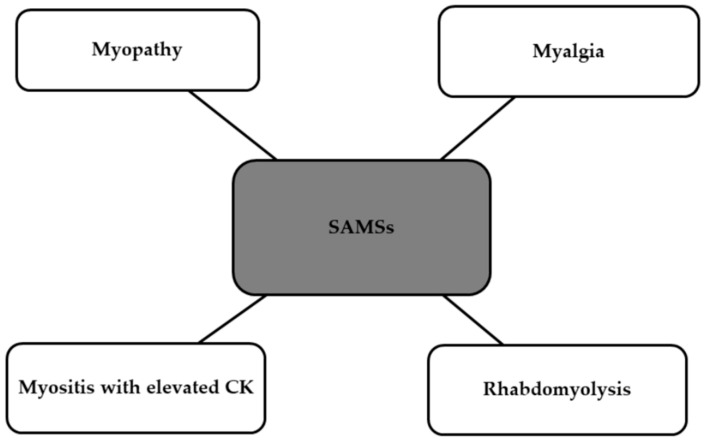
Side effects of statins classified as SAMSs [91]. SAMSs, statin-associated muscle symptoms; CK, creatinine kinase.

The American Heart Association Guidelines divide statin therapy into three categories based on the intensity: high-intensity, moderate-intensity, and low-intensity. Various ethnic or racial groups have demonstrated differing sensitivities to similar statin doses; for example, Asians may be more sensitive to the effects of statins [87]. A prospective, randomized, single-blind clinical trial was conducted to compare the lipid-lowering effect of statins (5 mg rosuvastatin), placebo, and six dietary supplements (fish oil, cinnamon, garlic, turmeric, plant sterols, red yeast rice). After 28 days, the decrease in LDL-C levels among these participants was examined, and the results were compared. The reduction in LDL-C percentage with rosuvastatin significantly exceeded that of all supplements and the placebo (the reduction in comparison to placebo was 35.2%) [88]. A meta-analysis published in JAMA demonstrated that both statin therapy and non-statin therapies, which regulate LDL receptor (LDLR) expression to lower LDL-C, show similar relative risks of major vascular events per change in LDL-C. Furthermore, lower levels of achieved LDL-C were linked with decreased rates of major coronary events. Furthermore, the European Society of Cardiology presented a paper stating that statin therapy in individuals without a history of CVD may lead to a 15% decrease in the risk of death from vascular causes for every 1 mmol/L reduction in LDL cholesterol [89,90]. Long-term use of statins can result in a range of adverse effects. Statin toxicity or intolerance mostly manifests as SAMSs (statin-associated muscle symptoms). These symptoms are shown in the Figure 3 below [91].

Another medication used to reduce LDL levels frequently mentioned in conjunction with statins is ezetimibe. Ezetimibe is an inhibitor of Niemann–Pick C1-Like 1 (NPC1L1), acting to block the absorption of cholesterol at the brush border of the small intestine, and it has proven an effective and usually well-received choice in treating hypercholesterolemia [91]. Incorporating ezetimibe with a statin has been demonstrated to help more patients achieve cholesterol levels recommended by guidelines and permits the utilization of lower doses of statins. This could be especially advantageous for individuals susceptible to the dose-related side effects of statins. Recent findings suggest that combinations of statins and ezetimibe are increasingly recognized for their ability to decrease the risk of major atherosclerotic events by an extent comparable to that observed with statins alone, even when achieving similar absolute reductions in LDL-C levels [92]. One of the studies analyzing the effect of ezetimibe with a statin was the IMPROVE-IT (Improved Reduction of Outcomes: Vytorin Efficacy International Trial). IMPROVE-IT was a double-blind, controlled trial involving 18,144 high-risk patients who had experienced stabilized ACS. Participants were randomly allocated to two groups: one receiving a combination of simvastatin 40 mg and ezetimibe 10 mg; and the other receiving simvastatin 40 mg alone. The occurrence of cardiovascular events among subjects was analyzed and proved to be reduced among patients taking simvastatin with ezetimibe [93,94]. A meta-analysis of 12 randomized, controlled trials was conducted to assess the effectiveness of ezetimibe in reducing LDL-C levels in individuals with ASCVD. The use of combination ezetimibe plus statin therapy demonstrated a greater absolute reduction in LDL-C levels compared to statin monotherapy. Therefore, it can be inferred that adding ezetimibe to statin therapy resulted in a modest additional reduction in LDL-C.

### 3.2. PCSK9 Inhibitors

In recent years, PCSK9 (proprotein convertase subtilisin/kexin type 9) inhibitors have emerged as a promising therapeutic option for managing ASCVD. PCSK9 is a protein produced primarily in the liver, where it plays a crucial role in regulating LDLR degradation. Elevated levels of PCSK9 lead to increased degradation of LDLRs, resulting in reduced LDL-C clearance from the bloodstream and elevated LDL-C levels [95]. PCSK9 inhibitors, like evolocumab (Repatha) and alirocumab (Praluent), are monoclonal antibodies that bind to PCSK9, preventing its interaction with LDLRs and subsequently increasing LDLR expression on the surface of hepatocytes [96,97]. This mechanism leads to enhanced LDL-C clearance and lowered LDL-C levels in the bloodstream [98]. Numerous clinical trials have demonstrated the efficacy of PCSK9 inhibitors in reducing LDL-C levels and improving cardiovascular outcomes in patients with ASCVD [99]. Trials such as FOURIER (Further Cardiovascular Outcomes Research With PCSK9 Inhibition in Subjects With Elevated Risk) and ODYSSEY OUTCOMES evaluated the cardiovascular benefits of evolocumab and alirocumab, respectively, in high-risk patients with established ASCVD. The FOURIER trial was a landmark study investigating the cardiovascular benefits of evolocumab in patients with established ASCVD. A total of 27,564 participants were enrolled and followed for a median of 2.2 years. In the FOURIER trial (multicenter, randomized, double-blind, placebo-controlled), treatment with evolocumab resulted in a significant reduction in LDL cholesterol levels by approximately 59%, compared to placebo. Additionally, the study found that evolocumab lowered the risk of major adverse cardiovascular events (MACE) by 15% over a median follow-up period of 2.2 years. These findings underscore the substantial cardiovascular benefits associated with PCSK9 inhibition in patients with established ASCVD [100,101]. In the ODYSSEY OUTCOMES trial (multicenter, randomized, double-blind, placebo-controlled trial), treatment with alirocumab led to a substantial reduction in LDL cholesterol levels by approximately 61% compared to placebo. Furthermore, alirocumab demonstrated a significant reduction in the risk of major adverse cardiovascular events (MACE), including MI, stroke, and cardiovascular death, by 15% over a median follow-up period of 2.8 years. These results provide robust evidence for the efficacy of PCSK9 inhibitors in reducing cardiovascular risk in patients with recent ACS [102,103]. Based on compelling evidence from clinical trials, major cardiovascular guidelines have incorporated PCSK9 inhibitors into their recommendations for ASCVD management. These guidelines endorse the use of PCSK9 inhibitors in specific patient populations, such as those with established ASCVD who require further LDL-C lowering despite maximally tolerated statin therapy, or in individuals with FH who cannot achieve LDL-C goals with traditional lipid-lowering therapies alone [100]. Overall, PCSK9 inhibitors have demonstrated a favorable safety profile in clinical trials, with adverse events similar to placebo. Common side effects include muscle pain, back pain, nasopharyngitis or headache [104]. One of the primary challenges associated with PCSK9 inhibitors is their cost, which has raised concerns regarding affordability and accessibility for patients and healthcare systems [105].

### 3.3. Bempedoic Acid

As an inhibitor of ATP citrate lyase (ACL), bempedoic acid offers a novel mechanism of action to reduce LDL-C levels and mitigate cardiovascular risk [106]. Bempedoic acid, previously known as ETC-1002, is an oral, once-daily medication that inhibits ACL, an enzyme involved in cholesterol synthesis. By blocking ACL, bempedoic acid reduces the production of cholesterol precursors, leading to decreased hepatic cholesterol synthesis and subsequent lowering of LDL-C levels [107]. Unlike statins, which act on HMG-CoA reductase, bempedoic acid operates upstream in the cholesterol biosynthesis pathway, offering an alternative therapeutic strategy for LDL-C reduction [108,109]. The efficacy of bempedoic acid in reducing LDL-C levels and improving cardiovascular outcomes has been demonstrated in several clinical trials [110]. The CLEAR Harmony and CLEAR Wisdom trials were randomized, double-blind, placebo-controlled phase 3 clinical trials investigating the safety and efficacy of bempedoic acid in patients with hypercholesterolemia and a high risk of CVD [111,112]. In the CLEAR Harmony trial, over 2200 patients were enrolled and randomized to receive either bempedoic acid or placebo in addition to maximally tolerated statin therapy. The trial demonstrated that treatment with bempedoic acid led to a significant reduction in LDL cholesterol levels by approximately 18% compared to placebo at 12 weeks [111]. Similarly, in the CLEAR Wisdom trial, which enrolled over 1800 patients, treatment with bempedoic acid resulted in a significant reduction in LDL cholesterol levels by approximately 17% compared to placebo at 12 weeks. Bempedoic acid was well tolerated in both trials, with adverse events similar to placebo [112]. The compelling findings from the trials have contributed to the regulatory approval of bempedoic acid by the United States Food and Drug Administration (FDA). In February 2020, the FDA approved bempedoic acid (Nexletol) as an adjunct to diet and maximally tolerated statin therapy for the treatment of adults with heterozygous familial hypercholesterolemia (HeFH) or established ASCVD who require additional LDL-C lowering [113,114]. Bempoedic acid is recommended in patients with ASCVD who require additional LDL-C lowering beyond statin therapy or in those who cannot tolerate statins [113]. Notably, bempedoic acid does not appear to increase the risk of adverse events such as muscle-related side effects or liver enzyme elevations typically associated with statin therapy [115]. Unfortunately, as newer medication, the cost of bempedoic acid may present a barrier to access for some patients [116].

### 3.4. Inclisiran

Inclisiran is a first-in-class small interfering RNA (siRNA) therapy designed to selectively target and inhibit PCSK9 messenger RNA (mRNA) in the liver. It specifically binds to N-acetylgalactosamine (GalNAc) and the asialoglycoprotein receptor (ASGPR) [117]. By silencing PCSK9 expression, inclisiran promotes the upregulation of hepatic LDLRs, leading to increased clearance of LDL-C particles from the bloodstream. This unique mechanism of action offers a promising approach to lowering LDL-C levels and reducing cardiovascular risk in patients with ASCVD [118]. Clinical trials, including the ORION program, have demonstrated the efficacy and safety of inclisiran in reducing LDL-C levels in patients with ASCVD or FH [119]. The ORION program consists of a series of clinical trials evaluating the efficacy, safety, and tolerability of inclisiran across diverse patient populations. The pivotal trials in the ORION program, including ORION-9 (Trial to Evaluate the Effect of Inclisiran Treatment on LDL-C in Subjects With HeFH), ORION-10 (Inclisiran for Participants With ASCVD and Elevated LDL-C), and ORION-11 (Inclisiran for Subjects With ASCVD or ASCVD-Risk Equivalents and Elevated LDL-C), demonstrated consistent and robust reductions in LDL-C levels with inclisiran therapy [120]. The ORION trials evaluated inclisiran’s efficacy when administered as a subcutaneous injection every 6 to 12 months. In these trials, inclisiran achieved substantial and durable reductions in LDL-C levels of up to 50%, surpassing those achieved with standard lipid-lowering therapies. Moreover, inclisiran exhibited a favorable safety profile, with minimal adverse effects observed across multiple studies [121,122]. Inclisiran (Leqvio) has received approval from both the FDA and the European Medicines Agency (EMA), in combination with dietary adjustments and the highest tolerated dosage of statin therapy, for adults diagnosed with HeFH or ASCVD necessitating further reduction in LDL cholesterol levels [123,124,125]. However, various factors should be taken into account when identifying suitable candidates for inclisiran therapy in clinical settings. They are listed in the Table 2 below [126].

### 3.5. Canakinumab

**Figure 4:**
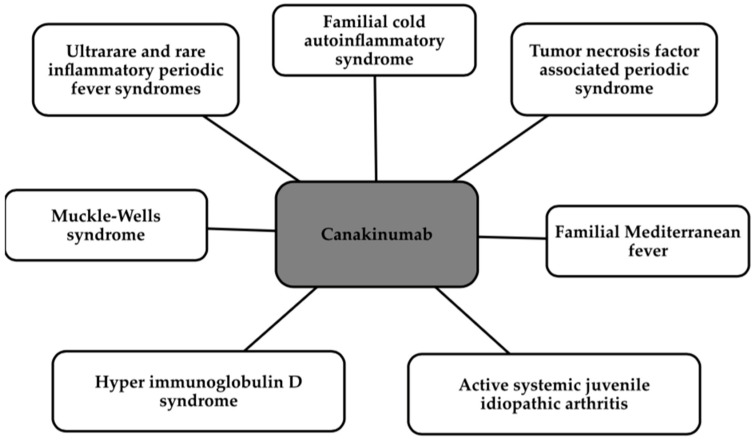
Indications for the use of Canakinumab [129].

Canakinumab is a monoclonal antibody which, by its action, neutralizes IL-1β signaling by blocking the inflammatory pathway [127,128]. Canakinumab directly inhibits IL-1β, while indirectly inhibiting matrix metalloproteinase (MMP), VCAM, intercellular adhesion molecule (ICAM), IL-6, and fibrinogen. The antibody has been approved by the FDA for the treatment of a number of conditions, which are shown in the Figure 4 below [129].

IL-1β is one of the first interleukins, which is an important mediator between intercellular communication in the immune system and is an important factor responsible for the immune response in atherosclerosis. IL-1β locally in the vessel wall causes the expression of adhesion molecules, cytokines, and chemokines and enhances the inflammatory response. There is evidence to suggest a proatherogenic effect of IL-1β in vascular smooth muscle through the induction of pro-inflammatory factors. As for its effect on the body, on the other hand, it mainly derives inflammation induced mainly by pro-inflammatory IL-6. Inflammation can be easily and quickly verified by rising blood levels of hsCRP, as its increase is clearly correlated with the pro-inflammatory effects of IL-1β and IL-6 [130]. One small study showed in patients with atherosclerosis and impaired glucose tolerance or type 2 diabetes a reduction in atherosclerotic plaque progression in the carotid arteries, reduced inflammation, and better perfusion of lower extremity musculature [131].

Regarding the effect of canakinumab, the randomized, double-blind, placebo-controlled CANTOS trial has been conducted [130,132,133]. This study included more than 10,000 patients with a history of MI and with hsCRP above 2 mg/L. The subjects were treated with hypolipemic therapy according to current recommendations, i.e., statins, and their cholesterol was 80 mg/dL. Participants received canakinumab—50,150, or 300 mg—or placebo subcutaneously every 3 months for 3.7 years [130,132,134].

The Canakinumab Anti-inflammatory Thrombosis Outcome Study (CANTOS) clinical trial looked at whether inhibition of IL-1β-induced inflammation could be effectively used for secondary prevention in high-risk patients in atherosclerotic ischemic incidents [130,134,135].

The study’s conclusions were a 15% reduction in the occurrence of three situations: non-fatal MI, fatal stroke, and a reduction in death overall from cardiovascular causes in those taking the 150 mg dose. The higher dose, 300 mg, had virtually the same effect as 150 mg, while the three-times-lower dose non-significantly reduced the situations studied. Thus, the conclusion is that a dose of at least 150 mg is protective against cardiovascular events in secondary prevention and is independent of the reduction in serum lipid levels. The use of canakinumab reduced hsCRP levels and thus generalized inflammation without reducing LDL cholesterol levels [130]. The most important positive results are mainly related to the reduction in the number of MIs, the reduction in the need for coronary revascularization, and the prevention of recurrent ischemic events in patients with both CKD and diabetes [130,132]. As for observed side effects, canakinumab increased the frequency of neutropenia, the risk of infections, and deaths from infections [134]. Above all, due to the anti-inflammatory effect of the drug, the greatest adverse effect was a decrease in the immunity of the recipient, thereby increasing the frequency of the above-mentioned infections [132]. The authors of the CANTOS study also believe that the antibody reduces atherothrombosis and causes many beneficial changes in the late stages of atherosclerosis, such as remodeling and collagen formation, but this still requires more research [136].

### 3.6. Tocilizumab

Tocilizumab is a recombinant humanized monoclonal antibody directed against the IL-6 receptor [137,138]. IL-6 is produced by T and B cells, fibroblasts, and monocytes. It is responsible for various processes such as T-cell activation, hemopoiesis, induction of hepatic acute phase protein synthase, and immunoglobulin secretion [139]. This drug is used in patients with rheumatoid arthritis or COVID-19 [140]. In patients with STEMI-type MI, an improvement in myocardial survival rate was observed after administration within 6 h of the incident IL-6 receptor antagonist. This showed a potential protective effect, but at the same time did not affect the final extent of the infarction. Thus, the study shows for the time being an uncertain effect on the cardiovascular system [141]. In one prospective cohort study, 28 patients with RA were observed. They received tocilizumab subcutaneously at a dose of 162 mg once a week or intravenously at 4 mg/kg b.w. every 4 weeks. After 3 months, the effect on the presence of underlying disease was evaluated, but we will focus on the effect on lipid parameters. The following parameters increased: total cholesterol, LDL-C, non-HDL-C (high-density lipoprotein cholesterol), and apo-B; while Lp(a) i and oxLDL decreased. However, no changes were noted in the levels of triglycerides, HDL-C and apo A-I. This study demonstrates the “lipid paradox”, i.e., an increase in lipid parameters with a concomitant decrease in inflammation in the body, which should be looked at in future studies [140]. It is noteworthy that in the CANTOS study mentioned with the previous drug, a reduction in cardiovascular risk was simultaneously associated with a reduction in IL-6 levels [142]. Thus, the possibility of tocilizumab should be looked at in connection with a non-traditional approach to cardiovascular risk factors and focusing on the presence of pro-inflammatory factors [143].

### 3.7. Janus Kinase Inhibitors

The Janus kinase (JAK) family of non-receptor protein-tyrosine kinases, represented by JAK1, JAK2, JAK3, and Tyk2, are responsible for signal transduction from membrane receptors to the cell nucleus via the JAK/STAT pathway, leading to the modulation of cell proliferation, differentiation, and apoptosis [144]. JAK inhibitors such as tofacitinib and baricitinib have found wide application in the treatment of inflammatory diseases such as rheumatoid arthritis (RA), systemic lupus erythematosus (SLE), and various dermatological conditions [145,146]. In SMC present in blood vessels, JAK/STAT participates in promoting proliferation associated with injury and angiotensin II-induced intracellular signaling. This suggests that JAK plays a significant role in the pathogenesis of numerous vascular diseases such as systemic hypertension, post-angioplasty restenosis, and atherosclerosis [147]. In rodent studies, it has been shown that blocking the activity or expression of JAK3 reduces the proliferation of smooth muscle cells induced by platelet-derived growth factor-BB (PDGF-BB) and inhibits injury-induced intimal hyperplasia [144]. Moreover, JAK3 plays a significant role in modulating the inflammatory response through the regulation of signal transducer and activator of transcription 3 (STAT3) activation, a key mediator of vascular responses to inflammation, and by participating in IL-6-dependent macrophage differentiation and IL-8-induced neutrophil chemotaxis. The recruitment of these cells to the injured blood vessel strongly correlates with subsequent neointimal formation [148,149,150]. The significant involvement of JAK in vascular remodeling processes suggests that JAK inhibitors may be utilized in the future as therapeutics for preventing the development of atherosclerosis and other vascular diseases. However, further research in this domain is necessary at present.

### 3.8. SGLT2 Inhibitors

The sodium-glucose cotransporter 2 (SGLT2) is the primary transporter of glucose located in the renal proximal tubule, responsible for transporting glucose from the renal tubule lumen into renal tubule epithelial cells [151]. The mechanism of action of SGLT2 inhibitors (SGLT2i) is based on inhibiting this protein, leading to a decrease in glucose concentration in the serum [151]. In addition to their main function, drugs from this group contribute to delaying the process of atherosclerosis by improving endothelial and VSMC dysfunction, preventing platelet activation, attenuating oxidative stress, and reducing inflammation [152]. Furthermore, SGLT2 inhibitors demonstrate a varied impact on metabolism, indirectly contributing to the slowing down of atherosclerosis processes. Meta-analyses of clinical trials conducted on patients treated with SGLT2i have indicated significant weight loss among the studied subjects, primarily attributed to calorie loss and the conversion of glucose metabolism to ketones and fatty acids. This process enhances fat utilization, ultimately resulting in weight reduction [153,154]. Additionally, SGLT2i reduce serum uric acid levels, which is hypothesized to act as a promoter of inflammatory processes and oxidative stress [155,156]. Another mechanism through which SGLT2i counteracts atherosclerosis is their influence on macrophages. Autophagy contributes to the removal of apoptotic macrophages from atherosclerotic plaques. Inhibiting autophagy renders macrophages more susceptible to cell death, exacerbating necrosis in advanced stages of atherosclerosis [157]. Studies conducted on mice have shown that canagliflozin could delay the progression of atherosclerosis by promoting macrophage autophagy. Canagliflozin promotes the expression of LC3II and the formation of autophagosomes, as well as enhancing cholesterol efflux from macrophages, resulting in lower lipid droplet concentrations in macrophages [158].

### 3.9. RAAS Inhibitors

Angiotensin II receptor blockers (ARBs) and angiotensin-converting enzyme inhibitors (ACEI) are widely used medications whose main function is to inhibit the renin-angiotensin–aldosterone system (RAAS). The RAAS is a system that plays an important role in maintaining the body’s electrolyte and fluid balance. However, it is a system closely associated with NADPH oxidase, which is an enzyme contributing to the generation of reactive oxygen species (ROS), the excessive production of which contributes to vascular injury and promotes atherogenesis. Additionally, the final effector of the RAAS, angiotensin II, is responsible for promoting inflammation, fibrosis, and acting directly on blood vessels to cause vasoconstriction [159]. These factors suggest that chronic activation of the RAAS may promote atherosclerosis and therapy with drugs inhibiting this system may lead to slowing down of atherosclerosis. Additionally, ARBs exhibit a broad spectrum of beneficial effects on vascular metabolism, including anti-inflammatory and antioxidative actions, which play a significant role in cardiovascular protection. Chronic therapy with ARBs reduces the onset of CVDs and decreases the likelihood of serious complications associated with these diseases, which is attributed to the hypotensive properties of these drugs [160].

### 3.10. GLP-1RA

GLP-1 receptor agonists (GLP-1RA) are new medications used in the treatment of type 2 diabetes. The mechanism of action of these drugs involves binding to the GLP-1 receptor (GLP-1R) and exerting incretin effects, which include glucose-dependent insulin secretion from pancreatic beta cells, inhibition of glucagon secretion from pancreatic alpha cells, and slowing of gastric emptying. GLP-1RA also acts centrally on neurons in the hypothalamus, inducing a feeling of satiety. Therapy with GLP-1RA reduces appetite, leading to weight loss, which indirectly contributes to their antiatherosclerotic effects [161]. GLP-1RA leads to a decrease in macrophage and monocyte accumulation in the arterial wall by inhibiting the inflammatory response in macrophages [162]. In studies conducted on mice, it was demonstrated that therapy with lixisenatide and liraglutide contributed to a significant reduction in the size of atheroma plaques [163]. Additionally, it has been demonstrated that GLP-1RA lead to a decrease in the level of CRP and pro-inflammatory cytokines and an increase in the level of adiponectin, which acts as an anti-inflammatory agent [164,165]. The anti-inflammatory action exerted by GLP-1RA indirectly affects the slowing of processes related to atherogenesis.

### 3.11. Antiplatelet Drugs

Platelets significantly contribute to the progression of atherosclerosis due to their interactions with endothelial cells of blood vessels and the release of inflammatory mediators, which initiate atherogenesis [166]. Antiplatelet drugs, through their anti-inflammatory effects and inhibitory influence on platelets, not only prevent thromboembolic events underlying the pathogenesis of ACS but may also exert a positive impact on slowing down processes associated with atherogenesis. Acetylsalicylic acid (ASA) acts by inhibiting cyclooxygenase-1 (COX-1), reducing the production of thromboxane A2 (TXA2), a vasoconstrictor that stimulates platelet aggregation [167]. Inhibiting COX-1 contributes to preventing cyclooxygenase-mediated cell proliferation and reducing the concentration of pro-inflammatory cytokines [168]. ASA also affects the reduction of IL release from platelets and is associated with the inhibition of endothelial dysfunction linked to the inflammatory process [169]. Moreover, ASA exhibits antioxidative properties and increases the availability of nitric oxide (NO), a natural vasodilator [170]. It has been demonstrated that ASA at a daily dose of 300 mg is associated with a significant reduction in the concentration of inflammatory markers such as IL-6 and CRP [168].

Cilostazol is an antiplatelet agent, acting through the inhibition of phosphodiesterase-3 (PDE-3) and subsequent elevation of cyclic adenosine monophosphate (cAMP) levels [171]. Its primary function is the inhibition of platelet aggregation, yet it also demonstrates anti-inflammatory, vasodilatory, and antioxidative properties, and improves lipid profile [172]. Administration of cilostazol leads to the phosphorylation of protein kinase A (PKA), resulting in the activation of endothelial nitric oxide synthase (eNOS), thereby increasing NO levels [173]. Elevated cAMP levels also contribute to the increased NO secretion by vascular endothelial cells, which is cAMP-dependent. Moreover, the increase in cAMP levels induces vessel dilation by stimulating the PKA-dependent activation of calcium-dependent potassium channels [174]. Moreover, cilostazol improves the lipid profile by upregulating lipoprotein lipase in adipose tissue and inhibiting PDE-3 in adipocytes [175,176]. Animal studies have demonstrated that cilostazol administration stimulates the expression of LDL receptor-related protein 1 (LRP1) in hepatocytes, which may significantly impact triglycerides and HDL-C improvement [177].

### 3.12. Sex Differences

Sex-related disparities in the efficacy and safety profiles of drugs with anti-inflammatory effects are increasingly recognized in the context of limiting the progression of ASCVD. Studies have indicated that canakinumab has shown promising results in reducing cardiovascular events and inflammation [178]. Additionally, statins, a cornerstone in ASCVD management, may exhibit differential effectiveness and adverse event rates between men and women, possibly influenced by variations in drug metabolism and hormone levels [179]. Estrogen, predominant in females, has been associated with potential cardioprotective effects, including anti-inflammatory properties and modulation of lipid metabolism, which might enhance the response to statins in women [180]. Conversely, androgens, more prevalent in males, might influence the metabolism and efficacy of certain anti-inflammatory medications. PCSK9 inhibitors have shown promising results in reducing cardiovascular events, yet emerging evidence suggests potential sex-specific responses to these agents, warranting further investigation [181]. Bempedoic acid, a novel cholesterol-lowering therapy, has demonstrated efficacy in both sexes; however, sex-based differences in tolerability and long-term cardiovascular outcomes require elucidation [122]. Similarly, newer agents like inclisiran, an RNA-targeted therapy, may exhibit sex-specific responses influencing their therapeutic benefits [182]. Moreover, while SGLT2 inhibitors and GLP-1 receptor agonists have shown cardiovascular benefits across sexes, the mechanisms underlying these effects and potential sex-related differences merit exploration [183]. Furthermore, antiplatelet drugs and RAAS inhibitors have well-established roles in ASCVD management, yet their efficacy and safety profiles may vary between men and women due to physiological and hormonal disparities. Understanding these sex-related differences in drug responses is crucial for optimizing treatment strategies and improving cardiovascular outcomes in both men and women [184].

### 3.13. Smoking Cessation

It is widely known that tobacco smoking is one of the most significant risk factors for cardiovascular events. The main mechanisms through which smoking contributes to the initiation of atherogenesis include endothelial dysfunction, induction of inflammation, elevation of pro-atherogenic lipid levels, and reduction in HDL levels [185]. Despite the widespread awareness of the harmful effects of smoking, patients often significantly struggle to quit smoking due to the highly addictive nature of nicotine. During the smoking cessation process, individuals may experience physical withdrawal symptoms such as difficulty concentrating, increased appetite, insomnia, restlessness, and anxiety [186,187]. To reduce withdrawal symptoms and thereby increase the likelihood of maintaining abstinence, pharmacological treatment can support patients. In recent years, substantial evidence has emerged, demonstrating the effectiveness of medications such as varenicline, cytisine, or antidepressant medications, among which bupropion exhibits the greatest efficacy in aiding smoking cessation [188,189,190]. Despite its effectiveness in alleviating nicotine withdrawal symptoms, varenicline exhibits adverse effects. In 2011, the FDA advised that varenicline may slightly increase the risk of cardiovascular events among patients with CVDs [189].

### 3.14. Proper Diet

The most desirable and least costly method to reduce cardiovascular risk is primary prevention, understood as primarily addressing obesity [191]. We have evidence that diet can affect the development of atherosclerosis [192]. Weight reduction affects lipidogram, systemic inflammation, and glucose metabolism, including a reduction in fasting glucose, hemoglobin A1c (HbA1c), and blood pressure, as well as disease progression [191,193]. Weight reduction through a calorie-restricted diet, in addition to weight change, improves insulin resistance, delays aging, or extends life expectancy [191]. A diet with reduced caloric intake improves atherosclerotic markers, reduces inflammatory markers such as hsCRP, nuclear factor kappa B, NFB, TNFα, and reduces superoxide production [193]. We know the factors that negatively affect the risk of cardiovascular factors, that is, poor hygiene and amount of sleep, poor diet, stress, little exercise, and poor air [191].

Lifestyle changes resulting in the reduction of risk factors should be implemented in patients’ lives through self-education or by preventive health practitioners to reduce the progression of atherosclerosis [194]. A proper dietary pattern is the main primary prevention of ASCVD [186]. The Table 3 shows the elements of a proper dietary pattern that will reduce the risk of ASCVD and the elements that will act adversely [195,196].

Due to the importance of diet in our lives, it is important to create good eating habits from the beginning in order to prevent inflammation in the body so that we can confidently inhibit the development of atherosclerosis [197].

## 4. Conclusions

Atherosclerosis is a typical vascular aging disease. Nonetheless, a number of variables might influence this process, increasing cardiovascular risk and resulting in a high rate of morbidity and death. In this review, we concentrated on the key molecular features of arteriosclerosis. We focused on the immune system’s involvement, dysfunctions, and impact on the progression of this disease. In light of atherosclerosis affecting over 230 million people globally, identifying biomarkers is essential for assessing risk and monitoring disease progression. Research has identified key biomarkers related to inflammation, such as the inflammasome, GDF-15, and immune cells impacting ox-LDL serum levels, particularly relevant in younger CAD patients for prognostic insights. Additionally, FGF-23 is elevated in a population with an increased risk of atherosclerotic lesions. Exosomes role in the development of CVDs were all regarded important. Exosomes—extracellular vesicles rich in miRNAs, lncRNAs, circRNAs—emerge as pivotal in understanding atherosclerosis due to their roles in cellular communication and disease mechanics. These findings underline the potential of exosomal miRNAs and non-coding RNAs as promising biomarkers for advancing the diagnosis, prognosis, and therapeutic targeting of atherosclerosis, highlighting the need for further research to integrate these biomarkers into clinical practice. Furthermore, because of the increasing number of people suffering for atherosclerotic CVD, we presented both well-known medicaments and newly discovered examples such as inclisiran, canakinumab, and Janus kinase inhibitors.

## Figures and Tables

**Table 1:** Pleiotropic effects of statins [84,85,86].

Effect	Mechanism
Anti-inflammatory	Reduction in CRP, IL-1β, TNF-α
	Decrease in leukocyte–endothelial cell adhesion
Plaque reduction/stabilization	Increased thickness of fibrous cap
	Macrocalcification
Reduced oxidative stress	Reduced ROS
Reduction of platelet aggregation	Decreased platelet reactivity
	Decreased TXA2 synthesis
Improved vascular tone	Increased NO
	Reduced SMC activation, proliferation

CRP, C-reactive protein; IL-1β, interleukin-1β; TNF, tumor necrosis factor; ROS, reactive oxygen species; TXA2, thromboxane A2; NO, nitric oxide; SMC, smooth muscle cell.

**Table 2:** Considerations for inclisiran prescription [126].

Indications for Initiating Inclisiran	Possible Impediments to Taking Inclisiran
Prior diagnosis of ASCVD or HeFH	Challenges in securing insurance coverage for inclisiran-based therapy
Inability or reluctance to use a self-injectable PCSK9 monoclonal antibody	Limited access to specialized care facilities due to geographical constraints or transportation issues
Need for further reduction in LDL cholesterol despite maximal statin therapy (with or without ezetimibe)	Patients belonging to demographic groups with limited safety data available for inclisiran use, such as those with CHF, liver disease, CKD, or pregnant women
Challenges with adhering to a bi-weekly dosing regimen of PCSK9 monoclonal antibodies;	High out-of-pocket expenses for specific patients depending on their insurance coverage
Potential consideration in patients experiencing statin-related side effects or demonstrating poor adherence to therapy, or encountering adverse effects from PCSK9 monoclonal antibodies	

ASCVD, atherosclerotic cardiovascular disease; HeFH, heterozygous familial hypercholesterolemia; CHF, congestive heart failure; CKD, chronic kidney disease.

**Table 3:** Food items that increase and decrease the risk of AVSCD [195,196].

Products That Decrease Risk of ASCVD	Products That Increase Risk of ASCVD
Fruits and non-starchy vegetables	Red and processed meats
Nuts	Refined carbohydrates
Legumes	Salt
Fish	Saturated fats
Vegetable oils	Added sugars
Whole grains	Ultra-processed foods

ASCVD—atherosclerotic cardiovascular disease.

## Data Availability

The data used in this article were sourced from materials mentioned in the References section.
